# Psychometric Evaluation of the Integral Nursing Leadership Scale Among Iranian Clinical Nurses: A Methodological Study

**DOI:** 10.1155/jonm/4342698

**Published:** 2025-10-12

**Authors:** Alireza Mirzaei, Pouya Mostafazadeh, Mehraban Shahmari, Nargess Ramazanzadeh, Sevda Gardashkhani, Reza Nemati-Vakilabad

**Affiliations:** ^1^Department of Nursing, Students Research Committee, School of Nursing and Midwifery, Ardabil University of Medical Sciences, Ardabil, Iran; ^2^Department of Emergency Nursing, School of Nursing and Midwifery, Ardabil University of Medical Sciences, Ardabil, Iran; ^3^Department of Medical-Surgical Nursing, School of Nursing and Midwifery, Ardabil University of Medical Sciences, Ardabil, Iran; ^4^Department of Nursing, Student Research Committee, School of Nursing and Midwifery, Tabriz University of Medical Sciences, Tabriz, Iran

**Keywords:** integral leadership, nursing, psychometric, reliability, validity

## Abstract

**Aim:**

This study aimed to assess the psychometric characteristics of the Persian version of the Integral Nursing Leadership Scale (INLS-P) within the Iranian clinical nurse population.

**Background:**

As nursing leadership roles gain importance, there is increasing demand for comprehensive leadership approaches addressing modern healthcare challenges. The INLS-P evaluates four critical dimensions: individual leadership qualities (developing personal competencies), individual performance (optimizing work execution), influencing organizational culture (shaping workplace values), and organizational excellence (driving quality outcomes)—all essential for holistic leadership development.

**Methods:**

Following COSMIN guidelines, this methodological study was conducted from June to August 2024. A multilevel approach was used: forward–backward translation, face and content validity, construct validity (confirmatory factor analysis), and reliability of the INLS-P were analyzed, respectively.

**Results:**

Our study revealed that the INLS-P demonstrates strong reliability across multiple metrics. Expert evaluations yielded the S-CVI/Ave score of 0.917 regarding content validity. For construct validity, confirmatory factor analysis revealed that the items loaded significantly on their respective factors, with factor loadings ranging from 0.48 to 0.91. Regarding reliability, the overall Cronbach's alpha was 0.924, indicating high internal consistency, while McDonald's omega was 0.931. Coefficient H reached 0.967, further supporting the scale's robustness. Mean interitem correlation averaged 0.427, reflecting solid relationships among items. The intraclass correlation coefficient (ICC) was also 0.917, confirming strong stability across the INLS-P.

**Conclusion:**

The findings indicate that the INLS-P has acceptable face validity, content validity, structural validity, and reliability when used to measure integral leadership qualities, particularly within nursing. This suggests that the INLS-P is a valid and reliable tool for assessing integral leadership skills in nursing.

## 1. Introduction

Nursing leadership faces unprecedented challenges in navigating modern healthcare's complexities—from workforce shortages and burnout epidemics to rapidly evolving care models [1]. Amid these pressures, effective nurse managers must not only align clinical teams with organizational strategy but also cultivate resilient work environments that sustain high-quality care [2, 3]. Contemporary research underscores leadership's dual mandate: empowering staff through resource optimization while driving transformational change in care delivery systems [4–6]. This delicate balance requires mastery of adaptive leadership strategies that reconcile competing priorities—staff well-being versus productivity metrics, protocol adherence versus innovation adoption, and immediate service demands versus long-term workforce development [7].

Nurses form the largest group of healthcare professionals worldwide and are essential for providing continuous and comprehensive healthcare services [8]. With the growing population, healthcare services and nursing care demand are increasing daily [9]. Improving the quality of nursing is paramount for enhancing nursing services and delivering more effective, efficient, and safe care to patients [10]. The current global crisis has emphasized challenges such as inadequate funding, a shortage of qualified nursing staff, and an aging population [7, 11]. The healthcare industry faces many challenges, and healthcare organizations need strong leadership to effectively tackle these issues and maintain high standards of patient care [12].

Healthcare organizations must provide high-quality care at an affordable cost and retain nursing staff [13]. To overcome these challenges, nurse managers need to adopt effective leadership styles [14]. The existence of good leadership practices provides safe and high-quality care and creates a high-quality work environment for nurses [7]. These leadership styles prioritize “people” or “relationships” and positively impact nurses, patients, and organizations, such as authentic and transformational leadership [15, 16]. In contrast, ineffective leadership styles employ destructive methods such as coercion, pressure, and control, which can undermine organizations [17]. These detrimental leadership styles can decrease work effectiveness and poor morale [18].

Improving leadership styles in health system organizations is vital for enhancing patient care and life expectancy [19]. Effective leadership correlates with nurse collaboration and positively influences teamwork, unit performance, and patient care quality [19, 20]. Satisfied nurses experience less fatigue and pressure, increasing patient satisfaction [21]. An integrated leadership approach is essential in complex healthcare environments, as no single style is universally effective [7]. This framework includes appreciative, relational, authentic, conscious, transformational, and compassionate leadership and focuses on leadership traits, individual achievement, and cultural impact [7, 22]. It facilitates efficient nursing management, improving patient services and organizational culture while increasing productivity and reducing absenteeism [23].

Developing a comprehensive assessment tool for integrated nursing leadership is crucial in light of the evolving challenges within the healthcare sector, such as demographic changes and increasing service demands [2, 7]. The Integral Nursing Leadership Scale, as highlighted by Cho & Choi, evaluates crucial leadership attributes across four key dimensions [7]. First, Individual Leadership Qualities assess the personal traits and competencies that define influential nursing leaders, such as emotional intelligence and communication skills. Second, Individual Performance focuses on nurses' accountability and professional growth, measuring how well they fulfill their roles and contribute to patient care. Third, the dimension of Influencing Organizational Culture examines a leader's ability to foster a positive work environment that encourages collaboration, innovation, and resilience among nursing staff, inspiring them to strive for excellence. Lastly, Organizational Excellence emphasizes the overall performance and quality of care within the healthcare organization, linking effective leadership to improved patient outcomes and operational success [7].

Additionally, it is imperative to validate the psychometric properties of the Persian version of the Integral Nursing Leadership Scale (INLS-P) to ensure its relevance across diverse cultural and clinical settings. This adaptation will empower nursing leaders to pinpoint specific areas for improvement and bolster the development of future leadership capabilities. By integrating various leadership styles and emphasizing these four dimensions, this tool aims to elevate nursing practices, enhance patient care standards, and nurture a culture of excellence. Ultimately, it contributes to establishing a more resilient healthcare system that can adeptly meet the demands of an increasingly complex landscape, thereby equipping nursing leaders with the confidence and skills to navigate the challenges.

## 2. Materials and Methods

### 2.1. Aim

This study aimed to assess the psychometric characteristics of the INLS-P within the Iranian clinical nurse population.

### 2.2. Design

The present study is a methodological study that carried out in the Ardabil province in northwestern Iran from June to August 2024. The psychometric evaluation was done in accordance with the Consensus-based Standards for the Selection of Health Measurement Instruments (COSMIN) guidelines [24].

### 2.3. Setting and Samples

The study encompassed all clinical nurses across various clinical wards in educational, treatment, and research hospitals in Ardabil province. Inclusion criteria necessitated a minimum of a bachelor's degree in nursing, at least 6 months of work experience in their respective units, consent to participate, and a minimum of 6 months of clinical experience. Participants who were unwilling to continue or left questions unanswered in the questionnaire were excluded from the study. For confirmatory factor analysis (CFA), a sample size of 200 to 400 individuals is recommended [25]. In this study, a convenience sampling method was employed to select a sample of 360 clinical nurses. The response rate for the survey was 90.8%, indicating a high level of participation. After excluding incomplete or inadequate responses, data from 327 participants were deemed valid and appropriate for further analysis, specifically CFA [26].

### 2.4. Integral Nursing Leadership Scale (INLS)

This scale was designed by Cho and Choi [7] to develop and evaluate an INLS for nurses. This scale consists of 30 items and four subscales: individual leadership qualities (9 items), individual performance (9 items), influencing organizational culture (7 items), and organizational excellence (5 items). Participants were asked to provide ratings for each item using a 6-point Likert scale, with options ranging from 1 (*strongly disagree*) to 6 (*strongly agree*). The INLS level is determined by the overall INLS score, with higher scores indicating greater integral nursing leadership.

### 2.5. Translation Procedure

**Figure 1:**
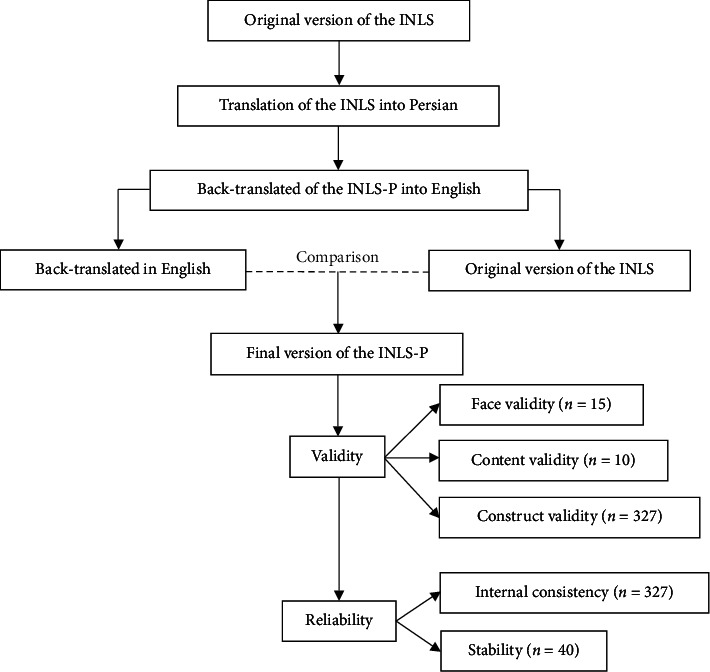
Flowchart of translation procedure and psychometric evaluation of the INLS-P.

The INLS-P was translated following the guidelines established by Wild et al. [27] and with the explicit permission of the scale's developer, Dr. JiSun Choi. The translation process involved the collaboration of two highly proficient translators, each with expertise in both English and Persian, who worked to translate the scale into Persian. The two translations were then thoroughly compared and adjusted to create a final version that accurately captured the original meaning. Following this, two additional translators, fluent in both languages, meticulously back-translated the Persian version into English to ensure accuracy and fidelity to the original concepts. The scale's developer then reviewed the translated version to guarantee that the core concepts were accurately conveyed. Subsequently, the translated INLS-P underwent rigorous validity and reliability testing, as depicted in Figure 1.

### 2.6. Psychometric Evaluation

#### 2.6.1. Face Validity

The INLS-P was administered to a group of 15 clinical nurses using purposive sampling as part of a qualitative evaluation to assess face validity. During this process, the nurses evaluated the items based on their perceived difficulty level, appropriateness, and wording ambiguity. To quantitatively measure face validity, the impact score method was used, employing a 5-point Likert scale ranging from “*not important*” (rated as 1) to “*quite important*” (rated as 5). Subsequently, the impact score was calculated using the following formula:

(1) $$\begin{array}{c} \text{Impact score}\left(\text{IS}\right)=\text{Frequency}\left(\%\right)\times \text{Importance}. \end{array}$$

An impact score greater than 1.5 indicates that the item is suitable for further analysis and will, therefore, be retained [28].

#### 2.6.2. Content Validity

We assessed the item-level content validity index (I-CVI) and scale-level content validity index (S-CVI) of the INLS-P. The I-CVI for each item of the INLS-P was evaluated by calculating the ratio of experts who rated it 3 or 4 to the total number of experts participating in the CVI assessment [29]. To calculate the S-CVI/Ave, we computed the average of the I-CVI values for all the items in the questionnaire. The I-CVI and S-CVI/Ave need to be above 0.78 and 0.90, respectively, to be considered acceptable [29, 30]. Moreover, we utilized CVI and multirater kappa statistics to address the potential for inflated values due to chance agreement [29]. To calculate modified kappa statistics, we initially determined the probability of chance agreement (*P*_*C*_) for each item using the following formula:

(2) $$\begin{array}{c} P_{C}=\frac{N!}{A!\left(N-A\right)!}\times 0.5^{N}. \end{array}$$

In this formula, *N* stands for the total number of experts on the panel, while A represents the number of experts concurring on good relevance (3 or 4) after calculating the I-CVI for all items. Following this, the modified kappa (*K*^∗^) was computed by inputting the numerical values of the probability of chance agreement (*P*_*C*_) and the I-CVI into the provided formula.

(3) $$\begin{array}{c} K^{*}=\frac{I-\text{CVI}-P_{C}}{1-P_{C}}. \end{array}$$

We assessed the *K*^∗^ values according to the following criteria: values equal to or less than 0.39 were categorized as poor, values ranging from 0.40 to 0.59 were deemed fair, values falling between 0.60 and 0.74 were evaluated as good, and values greater than 0.74 were considered excellent [29].

#### 2.6.3. Construct Validity

In the context of structural equation modeling (SEM), experts advocate for CFA as the preferred statistical approach over exploratory factor analysis (EFA) when the theoretical model aligns closely with the data structure [31]. Consequently, we employed CFA to assess the construct validity of the INLS-P.

It is important to note that there are no universally accepted criteria for determining goodness-of-fit indices (GFIs); instead, a diverse array of indices is essential as they can illuminate various aspects of the model's performance [32, 33]. In our analysis, we utilized several GFIs, including a Chi-square statistic (*χ*^2^) with a *p* value less than 0.05, a ratio of Chi-square to degrees of freedom (*χ*^2^/df) less than 3, the root mean square error of approximation (RMSEA) less than 0.08, the GFI greater than 0.90, the comparative fit index (CFI) greater than 0.90, the relative fit index (RFI) greater than 0.90, the incremental fit index (IFI) greater than 0.90, and a *p* value for the close fit (PCLOSE) greater than 0.05 [34–36].

We analyzed factor loadings to evaluate the relationships between observed variables (items) and their corresponding latent factors (subscales). A factor loading with an absolute value greater than 0.4 signifies a robust relationship, indicating that the observed variables effectively represent the underlying constructs [37]. Additionally, T-values were computed for each factor loading to ascertain their statistical significance. A T-value exceeding 1.96, corresponding to a significance level of 0.05, suggests that the observed variable significantly contributes to the latent factor, affirming the reliability of the factor loadings. This comprehensive analysis enhances our understanding of the measurement model's construct validity and ensures that the observed variables accurately reflect the intended latent constructs [38].

#### 2.6.4. Convergent and Discriminant Validity

To evaluate convergent and discriminant validity, several critical metrics were employed. composite reliability (CR) was calculated to assess the convergent validity of the indicators associated with each latent construct. A CR value exceeding 0.7 is generally considered indicative of acceptable reliability. Additionally, the average variance extracted (AVE) was measured, with values of 0.5 or higher signifying that the constructs account for more than half of the variance in their respective indicators, reinforcing convergent validity [39].

We analyzed the maximum shared squared variance (MSV) and average shared squared variance (ASV) to assess discriminant validity. A construct is deemed distinct if the MSV is less than the AVE and the ASV also falls below the AVE [39]. Furthermore, we calculated the Heterotrait–Monotrait (HTMT) ratio, with values below 0.85 indicating strong discriminant validity. This suggests that the constructs are sufficiently distinct, minimizing the risk of overlap [40].

#### 2.6.5. Reliability

To assess the INLS-P's internal consistency, we employed several key metrics. First, we calculated Cronbach's alpha coefficient (α) to evaluate the items' internal consistency, with values above 0.7 indicating acceptable reliability. Additionally, we utilized McDonald's omega (ω) as an alternative measure of reliability, particularly suited for multidimensional constructs, where values exceeding 0.7 are considered satisfactory. We also assessed Coefficient H to determine the scale's reliability, especially in the context of hierarchical structures, with values above 0.7 reflecting good reliability [41–46]. Furthermore, we calculated the mean interitem correlation (ρ) to gauge the average correlation between item pairs, where values typically ranging from 0.15 to 0.5 suggest good internal consistency, particularly when closer to the higher end of this range [47].

Lastly, the stability of the INLS-P across different raters or repeated measures was assessed by computing the intraclass correlation coefficient (ICC). For this assessment, 40 clinical nurses were selected through simple random sampling and measured at a two-week interval. An ICC value above 0.75 indicates good stability [48].

### 2.7. Data Analysis

Our analytical approach employed statistical protocols aligned with contemporary SEM standards. During preliminary screening, we identified multivariate outliers using Mahalanobis d-squared (*p* < 0.001)—a conservative threshold selected to minimize Type II errors while maintaining analytical integrity. Multivariate normality was assessed through Mardia's coefficient, with values > 8 indicating substantial kurtosis requiring covariance matrix adjustments [49].

For model evaluation, we adopted fit index thresholds rooted in methodological consensus: RMSEA < 0.08, balanced parsimony and model accuracy reflected adequate error approximation; CFI > 0.90, enhanced comparative improvement over baseline models; *χ*^2^/df < 3, mitigated Chi-square's sensitivity to sample size; GFI/IFI/RFI > 0.90, maintained > 90% covariance structure explanation. These criteria were operationalized through IBM SPSS AMOS Graphics, Version 24, to ensure robust model specification, particularly given our focus on nursing leadership constructs requiring precise parameter estimation. The PCLOSE > 0.05 threshold further confirmed that RMSEA's confidence intervals fell within acceptable bounds, reducing overfitting risks in clinical measurement models [34–36]. This multistep approach—from outlier removal (IBM SPSS Statistics for Windows, Version 24 (IBM Corp., Armonk, NY, USA)) to covariance modeling—was specifically designed to address healthcare leadership data's inherent complexity, where skewed distributions and multivariate relationships necessitate such methodological safeguards.

### 2.8. Ethical Considerations

The research study was conducted following the ethical guidelines and standards set by the Research Ethics Committees of Ardabil University of Medical Sciences, as indicated by approval ID: IR.ARUMS.REC.1403.102. The study strictly adhered to the ethical principles outlined in the revised Declaration of Helsinki, a set of internationally recognized guidelines for conducting medical research involving human subjects. Before participation, all study participants signed written informed consent and were assured that their personal information would be kept strictly confidential. Additionally, they were informed of their right to withdraw from the study at any point in time.

## 3. Results

### 3.1. Characteristics of the Participant

The study involved 327 participants with an average age of 31.96 years (SD = 4.81) and 6.41 years of working experience (SD = 4.51). Gender distribution was 42.5% male (*n* = 139) and 57.5% female (*n* = 188). Regarding marital status, 43.7% were single (*n* = 143) and 56.3% married (*n* = 184). Educationally, 63.0% held a Bachelor's degree (*n* = 206) and 31.5% a Master's degree (*n* = 103). Participants had primarily worked in medical wards (26.9%, *n* = 88) (Table 1).

### 3.2. Face Validity

The results indicated that most participants found the items relevant and reflective of their daily experiences in clinical settings. Several nurses suggested minor adjustments to improve clarity and reduce ambiguity. Overall, the qualitative feedback emphasized the importance of aligning the instrument with the realities faced by clinical nurses.

In the quantitative phase, all items of the measurement instrument achieved an impact score exceeding 1.5, indicating strong relevance and importance to the participants. Consequently, all the items were retained during this stage, confirming their face validity and importance for the study's goal (Table 2).

### 3.3. Content Validity

Table 2 displays the findings from the content validity evaluation of the INLS-P items. The S-CVI/Ave score of 0.917 suggests that the INLS-P is considered relevant at an acceptable standard. Furthermore, each item exhibited strong content validity (I-CVI > 0.78, *K*^∗^ > 0.74), indicating that experts consistently rated these items highly relevant. This strong consensus underscores the content validity of the items, affirming their appropriateness for assessing the intended constructs.

### 3.4. Descriptive Statistics of the INLS-P

The descriptive statistics for the INLS-P, based on 327 participants, indicated generally high scores in leadership dimensions. The mean scores ranged from 4.17 to 4.37, with standard deviations between 0.43 and 0.58. Skewness values suggested a tendency toward higher ratings, with floor effects ranging from 0.3% to 1.2% and ceiling effects from 0.9% to 7.3%. Overall, the mean score across all items was 4.35 (SD = 0.43). These findings reflected strong perceptions of leadership qualities and performance while revealing some response variability limitations (Table 3).

### 3.5. Construct Validity

**Figure 2:**
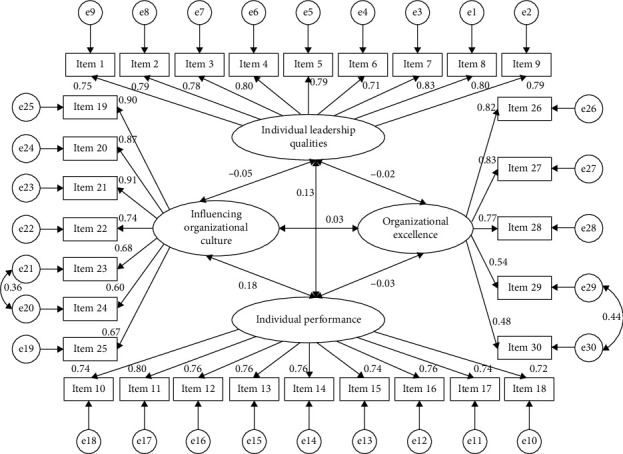
CFA measurement model of the INLS-P (*n* = 327).

The examination yielded a four-factor model comprising 30 items that accurately matched the INLS-P data (Figure 2). The CFA indicated that the model with the four factors had acceptable fit indices. The GFIs in the CFA showed *χ*^2^ = 1060.05, df = 302, *p* < 0.001, *χ*^2^/df = 1.717, RMSEA = 0.054, GFI = 0.902, CFI = 0.959, RFI = 0.906, NFI = 0.909, IFI = 0.960, PCFI = 0.826, and PCLOSE = 0.292. The factor loadings of the items ranged from 0.48 to 0.91, with T-values (S.E) ranging from 11.641 (0.50) to 24.263 (0.044) (*p* < 0.001).

### 3.6. Convergent and Discriminant Validity

Table 4 presents the results of the convergent and discriminant validity assessments for the INLS-P. The AVE values for all subscales exceeded 0.5, indicating that these values surpass the variance attributable to measurement error, confirming strong convergent validity. Moreover, the CR for all subscales was above 0.7, reflecting high internal consistency among the items within each subscale. These results suggested that the INLS-P effectively measured the intended constructs and was a valid instrument.

Regarding discriminant validity, the HTMT values for all dimensions were below 0.85, indicating adequate differentiation between the constructs. Additionally, the MSV and ASV values were generally lower than the AVE, reinforcing that the constructs are well-distinguished. These findings confirmed the INLS-P's discriminant validity and demonstrated its effectiveness in differentiating between various variables.

### 3.7. Reliability

The reliability analysis of the INLS-P yielded promising results across various dimensions. The Cronbach's alpha (α) values ranged from 0.812 to 0.899, indicating high internal consistency. McDonald's omega (ω) coefficients varied between 0.822 and 0.906, further supporting the scale's reliability. The Coefficient H (H) values were high, with a maximum of 0.940, reflecting robust reliability across the constructs. The mean interitem correlation (ρ) values varied between 0.401 and 0.421, which reflects a strong relationship among items within each construct. The overall Cronbach's alpha was 0.924 for the entire scale, and the McDonald's omega coefficient was 0.931. The Coefficient H for the total scale reached 0.967, indicating excellent reliability. The mean interitem correlation for the total scale was 0.427, further emphasizing the coherence among items (Table 5).

The ICC values for the subscales ranged from 0.818 to 0.940, with the overall ICC being 0.917 (95% CI: 0.873–0.949), indicating strong stability among items across the entire instrument (Table 5).

## 4. Discussion

Given the dynamic and intricate nature of healthcare systems, there is a growing need for a new and thorough approach to developing more effective leadership [50–52]. The psychometric validation of the INLS-P yielded robust evidence for its utility in clinical leadership assessment. Key findings demonstrated excellent reliability (Cronbach's *α* = 0.812–0.899; ICC = 0.818–0.940) and strong construct validity through a four-factor model (RMSEA = 0.054, CFI = 0.959), aligning with contemporary leadership frameworks emphasizing transformational leadership, staff empowerment, resource optimization, and quality-driven care. Notably, the scale's test–retest stability (ICC = 0.917) and discriminant validity (HTMT < 0.85) position it as a critical tool for addressing three pressing challenges in nursing leadership: targeted competency development, the subscale reliability metrics (e.g., *ω* = 0.906 for transformational leadership) enable precise identification of leadership gaps during annual competency evaluations; evidence-based promotion criteria, high ICC values (0.873–0.949) support using the INLS-P as an objective metric for leadership advancement decisions, reducing subjective bias, and crisis-readiness benchmarking, the strong convergent validity (AVE > 0.5) allows health systems to establish baseline leadership profiles for pandemic preparedness, particularly given participants' demographic profile (mean age 31.96 ± 4.81 years, 63% Bachelor's holders) reflecting frontline nurse leaders.

The INLS comprises 30 items distributed among four subscales. The first subscale, “individual leadership qualities,” consisting of 9 items, evaluates essential personal attributes and characteristics for effective nursing leadership, such as emotional intelligence, decision-making skills, adaptability, and the ability to inspire and motivate others. These qualities are crucial for nurses to navigate complex clinical environments and cultivate positive team relationships. The second subscale, “individual performance,” also with 9 items, assesses a nurse's effectiveness and efficiency in their role, including clinical skills, adherence to best practices, and achieving patient outcomes. High scores in this subscale indicate consistently meeting or exceeding performance expectations, which is essential for providing quality patient care. The “influencing organizational culture” subscale (7 items) evaluates a nurse's performance effectiveness and efficiency, encompassing clinical skills, adherence to best practices, and achieving patient outcomes. High scores here also indicate consistently meeting or exceeding performance expectations, which is crucial for delivering quality patient care. Lastly, the “organizational excellence” subscale (5 items) focuses on the broader organizational outcomes influenced by nursing leadership, including aspects such as the quality of care, patient satisfaction, and operational efficiency. High scores in this subscale reflect a nurse's contribution to achieving organizational goals and maintaining high standards of practice, ultimately leading to improved patient outcomes and organizational success [7].

Our results indicated that most participants found the items relevant and reflective of their daily clinical experiences. This qualitative feedback highlighted the importance of aligning the instrument with the realities faced by clinical nurses. Several nurses suggested minor adjustments to improve clarity and reduce ambiguity, demonstrating an active engagement with the instrument. In contrast, the original study [7] did not report participant feedback regarding face validity, which limits understanding of how well the items resonate with clinical nurses. Additionally, all items in our study achieved an impact score exceeding 1.5, confirming their strong relevance and importance. This contrasts with the original study, which focused more on quantitative assessments without elaborating on qualitative insights [7].

Regarding content validity, our evaluation yielded an S-CVI/Ave score of 0.917, while the original study reported a higher score of 0.96 [7]. Both studies demonstrated strong content validity, with our items showing I-CVI values greater than 0.78, consistent with the original study's findings of I-CVI values at 0.84. This suggests that while our items are relevant and appropriate for assessing intended constructs, experts rated the original study's items as slightly more relevant. Furthermore, the original study revised three items based on expert opinions to enhance clarity [7]. In contrast, our study did not necessitate item removal but emphasized the importance of qualitative feedback to refine the instrument.

Both studies successfully identified a four-factor model comprising 30 items, confirming the instrument's robustness across different analytical approaches. Our findings, derived from CFA, demonstrated strong fit indices and significant factor loadings, reinforcing the instrument's validity in measuring the intended constructs. The original study's EFA provided foundational insights into the factor structure, highlighting the appropriateness of the items based on their loadings and variance explained. The original study's use of EFA allowed for a more exploratory approach, revealing nuanced insights such as double-loading items and conceptual reallocations, which are crucial for refining the instrument [7]. In contrast, our CFA focused on confirming the predefined structure, which adds a layer of validation but may lack the exploratory depth captured in the original study.

In evaluating the reliability of the INLS-P, both our findings and those of the original study [7] demonstrate strong internal consistency, though there were notable differences in specific metrics. Our study reported a Cronbach's alpha of 0.924 for the entire scale, which, while robust, was slightly lower than the 0.98 reported in the original study. This suggests that while our scale maintains high reliability, the original study may reflect a more consistent internal structure across its sample. For various dimensions, our Cronbach's alpha values ranged from 0.812 to 0.899, indicating good reliability, albeit lower than the original study's range of 0.93 to 0.95. Similarly, McDonald's omega coefficients further support the reliability of both studies. Our findings yielded an omega of 0.931 for the entire scale, compared to 0.98 in the original study. While both scales are reliable, the original study presents a slightly stronger internal consistency. The Coefficient H in our study reached an impressive 0.967, reflecting excellent reliability across constructs. However, the original study did not provide specific values for this metric, making direct comparison difficult. Nevertheless, our high Coefficient H suggests that the items within our scale are well-aligned and consistently measure the intended constructs. The mean interitem correlation (ρ) in our study averaged 0.427, indicating a strong relationship among items. In contrast, the original study provided a range for corrected item-total correlations from 0.63 to 0.84, highlighting a solid interitem coherence but making it challenging to compare with our findings directly. Lastly, our study's ICC was 0.917, indicating strong stability among items across the entire instrument. The original study did not report ICC values, limiting our ability to assess this reliability aspect in comparison.

### 4.1. Limitations

However, our study had several limitations. Firstly, our study employed a cross-sectional design, which limits the ability to draw causal inferences about the relationships between variables. Additionally, the reliance on self-reported measures may introduce response biases, as participants might provide socially desirable answers rather than accurate reflections of their experiences. Furthermore, the absence of longitudinal data restricts our ability to assess changes in the constructs over time. Although our sample size was substantial, the demographic diversity may have been limited, potentially affecting the generalizability of our findings to broader populations. Despite these limitations, our study exhibited several strengths. Firstly, we utilized a robust sample size, enhancing our findings' generalizability and providing a solid basis for reliability analysis. Secondly, we conducted a comprehensive reliability assessment by employing multiple metrics to evaluate the INLS-P's internal consistency thoroughly. Thirdly, our analysis covered various dimensions of nursing leadership, allowing for a nuanced understanding of the constructs measured by the INLS-P. Moreover, the strong mean interitem correlation values indicate a solid relationship among items, reinforcing the coherence and relevance of the items within each dimension. Finally, our findings align closely with the existing literature, further validating the INLS-P and its applicability in diverse contexts.

## 5. Conclusions

The validation of the INLS-P underscores its vital role as a robust assessment tool for integral nursing leadership. It enables healthcare institutions to systematically identify leadership strengths and tailor development programs that address competency gaps. Practical applications include enhancing clinical decision-making through data-driven competency assessments, improving patient satisfaction scores by linking leadership competencies to care quality metrics, and adapting the scale to diverse healthcare systems, from urban hospitals to rural clinics, while preserving psychometric integrity. The instrument's structure facilitates improved nurse–physician collaboration, reduces medication errors through targeted interventions, and supports the implementation of culturally contextualized leadership frameworks. Future efforts should focus on longitudinal studies to track leadership development outcomes and cross-cultural validation across healthcare models. Ultimately, this will strengthen the scale's ability to promote evidence-based practices that enhance patient care quality and organizational effectiveness across various clinical environments.

## Figures and Tables

**Table 1:** Baseline characteristics of the participants (*n* = 327).

**Variable**	**Category**	**Mean ± SD**	

Age (year)		31.96 ± 4.81	
Working experience (year)		6.41 ± 4.51	

		**No.**	**Percentage**

Gender	Male	139	42.5
	Female	188	57.5

Marital status	Single	143	43.7
	Married	184	56.3

Education level	Bachelor's degree	206	63.0
	Master's degree	103	31.5

Ward	Medical	88	26.9
	Surgical	64	19.6
	Emergency	50	15.3
	ICU	38	11.6
	Others	87	26.6

**Table 2:** Results of the face and content validity of the INLS-P (*n* = 10).

Item	IS	A	I-CVI	*P*_*C*_	*K* ^∗^
1	3.4	8	0.8	0.044	0.79
2	4.7	10	1	0.001	1
3	3.8	9	0.9	0.01	0.89
4	2.5	8	0.8	0.044	0.79
5	3.2	10	1	0.001	1
6	2.7	10	1	0.001	1
7	3.4	10	1	0.001	1
8	2.5	8	0.8	0.044	0.79
9	3.4	9	0.9	0.01	0.89
10	2.7	8	0.8	0.044	0.79
11	2.7	10	1	0.001	1
12	2.5	8	0.8	0.044	0.79
13	3.4	8	0.8	0.044	0.79
14	3.7	10	1	0.001	1
15	2.7	9	0.9	0.01	0.89
16	3.8	10	1	0.001	1
17	4.7	9	0.9	0.01	0.89
18	4.8	10	1	0.001	1
19	3.4	10	1	0.001	1
20	3.8	9	0.9	0.01	0.89
21	2.7	9	0.9	0.01	0.89
22	3.2	10	1	0.001	1
23	3.7	10	1	0.001	1
24	3.4	8	0.8	0.044	0.79
25	2.7	10	1	0.001	1
26	3.4	10	1	0.001	1
27	2.5	8	0.8	0.044	0.79
28	3.4	9	0.9	0.01	0.89
29	2.7	8	0.8	0.044	0.79
30	4.7	9	0.9	0.01	0.89

*Note:* A, number agreeing on good relevance; *P*_*C*_, probability of a chance occurrence; *K*^∗^, kappa-designating agreement on relevance.

Abbreviations: I-CVI, item content validity index; IS, impact score.

**Table 3:** Descriptive statistics of the INLS-P (*n* = 327).

Dimensions	No. of item	Mean ± SD	Skewness	Kurtosis	Floor effect (%)	Ceiling effect (%)
Individual leadership qualities	9	4.30 ± 0.58	−1.12	−1.27	1 (0.3)	3 (0.9)
Individual performance	9	4.37 ± 0.53	−1.08	−1.44	1 (0.3)	4 (1.2)
Influencing organizational culture	7	4.17 ± 0.51	−1.78	−1.63	2 (0.6)	7 (2.1)
Organizational excellence	5	4.36 ± 0.48	−1.84	−1.85	4 (1.2)	24 (7.3)
Total	30	4.35 ± 0.43	−1.74	−1.61	2 (0.6)	2 (0.6)

**Table 4:** Results of the convergent and discriminant validity of the INLS-P (*n* = 327).

Latent factors	CR	AVE	MSV	ASV	HTMT			
					1	2	3	4
1. Individual leadership qualities	0.924	0.613	0.016	0.006	—			
2. Individual performance	0.923	0.602	0.032	0.016	0.488	—		
3. Influencing organizational culture	0.916	0.672	0.032	0.012	0.461	0.332	—	
4. Organizational excellence	0.840	0.505	0.002	0.001	0.478	0.512	0.401	—

*Note:* Numbers 1–4 in the title row represent the numbered variables in the first column. ASV, average shared squared variance; HTMT, Heterotrait–Monotrait ratio of correlation; MSV, maximum shared squared variance.

Abbreviations: AVE, average variance extracted; CR, composite reliability.

**Table 5:** Results of the reliability of the INLS-P (*n* = 327).

Dimensions	*α*	*ω*	*H*	*ρ*	ICC (95% CI)
Individual leadership qualities	0.812	0.822	0.933	0.412	0.932 (0.908–0.961)
Individual performance	0.868	0.873	0.917	0.401	0.871 (0.779–0.933)
Influencing organizational culture	0.882	0.895	0.940	0.421	0.818 (0.656–0.901)
Organizational excellence	0.899	0.906	0.866	0.417	0.940 (0.908–0.966)
Total	0.924	0.931	0.967	0.427	0.917 (0.873–0.949)

*Note:* α, Cronbach's alpha; ω, McDonald's omega coefficient; H, Coefficient H; *ρ*, mean interitem correlation.

Abbreviations: CI, confidence interval; ICC, intraclass correlation coefficient.

## Data Availability

The data that support the findings of this study are available from the corresponding author upon reasonable request.

## References

[1] Cummings G. G., Lee S., Tate K. (2021). The Essentials of Nursing Leadership: A Systematic Review of Factors and Educational Interventions Influencing Nursing Leadership. *International Journal of Nursing Studies*.

[2] Alsadaan N., Salameh B., Reshia F. (2023). Impact of Nurse Leaders Behaviors on Nursing Staff Performance: A Systematic Review of Literature. *Inquiry*.

[3] Harmoinen M., Suominen T. (2020). Realizing Appreciative Management From the Viewpoint of First‐Line Managers in Social and Health Care. *Scandinavian Journal of Caring Sciences*.

[4] Alhalal E., Alharbi J. F., Alharbi S. T. (2024). Impact of Authentic Leadership on Nurses’ Well‐Being and Quality of Care in the Acute Care Settings. *Journal of Nursing Scholarship*.

[5] Labrague L. J. (2024). Relationship Between Transformational Leadership, Adverse Patient Events, and Nurse-Assessed Quality of Care in Emergency Units: The Mediating Role of Work Satisfaction. *Australasian Emergency Care*.

[6] Labrague L. J., Al Sabei S. D., AbuAlRub R. F., Burney I. A., Al Rawajfah O. (2021). Authentic Leadership, Nurse‐Assessed Adverse Patient Events and Quality of Care: The Mediating Role of Nurses’ Safety Actions. *Journal of Nursing Management*.

[7] Cho S. M., Choi J. (2024). Integral Leadership in Nursing: Development and Psychometric Validation of a Korean Version of the Integral Nursing Leadership Scale. *International Journal of Nursing Studies*.

[8] Kulju E., Jarva E., Oikarinen A., Hammarén M., Kanste O., Mikkonen K. (2024). Educational Interventions and Their Effects on Healthcare Professionals’ Digital Competence Development: A Systematic Review. *International Journal of Medical Informatics*.

[9] Gunawan J. (2023). Exploring the Future of Nursing: Insights From the ChatGPT Model. *Belitung Nursing Journal*.

[10] Lucas P., Jesus É., Almeida S., Araújo B. (2023). Relationship of the Nursing Practice Environment With the Quality of Care and Patients’ Safety in Primary Health Care. *BMC Nursing*.

[11] Peters M. (2023). Time to Solve Persistent, Pernicious and Widespread Nursing Workforce Shortages. *International Nursing Review*.

[12] Ezzati F., Mosadeghrad A. M., Jaafaripooyan E. (2023). Resiliency of the Iranian Healthcare Facilities Against the Covid-19 Pandemic: Challenges and Solutions. *BMC Health Services Research*.

[13] Shen H. C., Li C. C., Yeh S. C. J. (2024). Do Hospitals Attaining a Public Recognition for Treating Nurses Fairly Deliver Better‐Quality Health Care? Evidence From Cross‐Sectional Analysis of California Hospitals. *Journal of Advanced Nursing*.

[14] Mohamed S. I., Nabway Z. M., Saleh M. S. (2023). The Correlation Between First–Line Nurse Managers’ Leadership Style and Staff Nurses’ Structural Empowerment and Work Engagement. *Alexandria Scientific Nursing Journal*.

[15] Al Sabei S. D., Ross A. M. (2023). The Relationship Between Nursing Leadership and Patient Readmission Rate: A Systematic Review. *Canadian Journal of Nursing Research*.

[16] Pattison N., Corser R. (2023). Compassionate, Collective or Transformational Nursing Leadership to Ensure Fundamentals of Care are Achieved: A New Challenge or Non‐Sequitur?. *Journal of Advanced Nursing*.

[17] Wolor C. W., Ardiansyah A., Rofaida R., Nurkhin A., Rababah M. A. (2022). Impact of Toxic Leadership on Employee Performance. *Health Psychology Research*.

[18] Shih F. C., Yeh S. J., Hsu W. L. (2023). Abusive Supervision and Employee Well-Being of Nursing Staff: Mediating Role of Occupational Stress. *Journal of Advanced Nursing*.

[19] Faal Sanati S., Barfar E., Payandeh A., Khammarnia M. (2024). Relationship Between the Leadership Styles of Nursing Managers and Teamwork of Nurses in University Hospitals of Mashhad and Zahedan, Iran. *Journal of health research in community*.

[20] Laschinger H. K. S., Read E. A. (2016). The Effect of Authentic Leadership, person-job Fit, and Civility Norms on New Graduate Nurses’ Experiences of Coworker Incivility and Burnout. *The Journal of Nursing Administration: The Journal of Nursing Administration*.

[21] Zaghini F., Fiorini J., Piredda M., Fida R., Sili A. (2020). The Relationship Between Nurse Managers’ Leadership Style and Patients’ Perception of the Quality of the Care Provided by Nurses: Cross Sectional Survey. *International Journal of Nursing Studies*.

[22] Baumhover N. (2020). Integrative Leadership in a Bachelor of Science in Nursing–Integrative Health Program. *Nurse Leader*.

[23] Lui J. N. M., Andres E. B., Johnston J. M. (2024). How do Organizational Culture and Leadership Style Affect Nurse Presenteeism and Productivity?: A Cross Sectional Study of Hong Kong Acute Public Hospitals. *International Journal of Nursing Studies*.

[24] Mokkink L. B., Terwee C. B., Patrick D. L. (2010). The COSMIN Checklist for Assessing the Methodological Quality of Studies on Measurement Properties of Health Status Measurement Instruments: An International Delphi Study. *Quality of Life Research*.

[25] Jackson D. L. (2001). Sample Size and Number of Parameter Estimates in Maximum Likelihood Confirmatory Factor Analysis: A Monte Carlo Investigation. *Structural Equation Modeling: A Multidisciplinary Journal*.

[26] Hox J. J. (2021). Confirmatory Factor Analysis. *The Encyclopedia of Research Methods in Criminology and Criminal Justice*.

[27] Wild D., Grove A., Martin M. (2005). Principles of Good Practice for the Translation and Cultural Adaptation Process for Patient-Reported Outcomes (PRO) Measures: Report of the ISPOR Task Force for Translation and Cultural Adaptation. *Value in Health*.

[28] Polit D. F., Yang F. M. (2016). Measurement and the Measurement of Change: A Primer for the Health Professions. *Wolters Kluwer Philadelphia*.

[29] Polit D. F., Beck C. T., Owen S. V. (2007). Is the CVI an Acceptable Indicator of Content Validity? Appraisal and Recommendations. *Research in Nursing & Health*.

[30] Polit D. F., Beck C. T. (2006). The Content Validity Index: Are You Sure You Know What’s Being Reported? Critique and Recommendations. *Research in Nursing & Health*.

[31] Kyriazos T. A. (2018). Applied Psychometrics: Sample Size and Sample Power Considerations in Factor Analysis (EFA, CFA) and SEM in General. *Psychology*.

[32] Bowen N. K., Guo S. (2011). *Structural Equation Modeling*.

[33] Nemati-Vakilabad R., Khoshbakht-Pishkhani M., Maroufizadeh S., Javadi-Pashaki N. (2024). Translation and Validation of the Persian Version of the Perception to Care in Acute Situations (PCAS-P) Scale in Novice Nurses. *BMC Nursing*.

[34] Byrne B. M. (2013). *Structural Equation Modeling With Mplus: Basic Concepts, Applications, and Programming*.

[35] Floyd F. J., Widaman K. F. (1995). Factor Analysis in the Development and Refinement of Clinical Assessment Instruments. *Psychological Assessment*.

[36] Hashemian Moghadam A., Nemati-Vakilabad R., Imashi R. (2024). The Psychometric Properties of the Persian Version of the Innovation Support Inventory (ISI-12) in Clinical Nurses: A Methodological Cross-Sectional Study. *BMC Nursing*.

[37] Marsh H. W., Muthén B., Asparouhov T. (2009). Exploratory Structural Equation Modeling, Integrating CFA and EFA: Application to Students’ Evaluations of University Teaching. *Structural Equation Modeling: A Multidisciplinary Journal*.

[38] Kline P. (2014). *An Easy Guide to Factor Analysis*.

[39] Fornell C., Larcker D. F. (1981). Evaluating Structural Equation Models with Unobservable Variables and Measurement Error. *Journal of Marketing Research*.

[40] Henseler J., Ringle C. M., Sarstedt M. (2015). A New Criterion for Assessing Discriminant Validity in Variance-Based Structural Equation Modeling. *Journal of the Academy of Marketing Science*.

[41] Dunn T. J., Baguley T., Brunsden V. (2014). From Alpha to Omega: a Practical Solution to the Pervasive Problem of Internal Consistency Estimation. *British Journal of Psychology*.

[42] Kalkbrenner M. T. (2023). Alpha, Omega, and H Internal Consistency Reliability Estimates: Reviewing These Options and when to Use Them. *Counseling Outcome Research and Evaluation*.

[43] Mayers A. (2013). Introduction to Statistics and SPSS in Psychology.

[44] McNeish D. (2018). Thanks Coefficient Alpha, We’Ll Take It From Here. *Psychological Methods*.

[45] Spiliotopoulou G. (2009). Reliability Reconsidered: Cronbach’s Alpha and Paediatric Assessment in Occupational Therapy. *Australian Occupational Therapy Journal*.

[46] Streiner D. L., Norman G. R., Cairney J. (2015). *Health Measurement Scales: A Practical Guide to Their Development and Use*.

[47] Clark L. A., Watson D. (2016). Constructing Validity: Basic Issues in Objective Scale Development.

[48] Koo T. K., Li M. Y. (2016). A Guideline of Selecting and Reporting Intraclass Correlation Coefficients for Reliability Research. *Journal of chiropractic medicine*.

[49] Vinzi V. E., Chin W. W., Henseler J., Wang H. (2009). Perspectives on Partial Least Squares. *Handbook of Partial Least Squares: Concepts, Methods and Applications*.

[50] Mirzaei A., Imashi R., Saghezchi R. Y., Jafari M. J., Nemati-Vakilabad R. (2024). The Relationship of Perceived Nurse Manager Competence with Job Satisfaction and Turnover Intention Among Clinical Nurses: An Analytical cross-sectional Study. *BMC Nursing*.

[51] Noorzad O. S. (2005). *The Integral Leadership Quest: An Integrated Theoretical and Practice-Based Approach and Framework for Organizational Leadership*.

[52] Rosser E., Westcott L., Ali P. A. (2020). The Need for Visible Nursing Leadership During COVID‐19. *Journal of Nursing Scholarship*.

